# Regional differences, distribution dynamics, and convergence of multidimensional food security levels in the Yangtze river economic belt

**DOI:** 10.1371/journal.pone.0340775

**Published:** 2026-02-19

**Authors:** Jing Cheng, Yaning Liu

**Affiliations:** School of Humanities and Social Sciences, Jiangsu University of Science and Technology, Zhenjiang, Jiangsu, China; USTC: University of Science and Technology of China, CHINA

## Abstract

Food security is a key foundation for the Yangtze River Economic Belt’s high-quality development. Using a multidimensional food security evaluation framework that encompasses quantity, quality, ecology, and capacity, this paper analyzes 131 prefecture-level cities from 2009 to 2022 through Gini coefficient decomposition, kernel density estimation, and convergence modeling. The study reveals an overall steady rise in food security, characterized by shrinking but still significant regional disparities and increasing coordination. The region’s distribution dynamics exhibit an olive-shaped structure, characterized by being “big in the middle and small at the ends.” Further analysis reveals that the midstream region converges the fastest due to policy alignment and technology diffusion, whereas the downstream region, hindered by urbanization and market segmentation, experiences the “high β coefficient - low convergence speed” paradox. The key drivers of divergent convergence paths are policy heterogeneity and misallocated resources. Based on these findings, differentiated governance strategies are proposed to promote integrated food security and ecological protection.

## 1. Introduction

Food security is a fundamental strategic issue. It is tied to livelihood, stability, sovereignty, and security. It has always played a central role in national governance. The 20th CPC National Congress report urges, as a modernization strategy, to “accelerate the construction of a strong agricultural country.” It also highlights the importance of building an all-chain food security system and strengthening the foundation of food security. The report aims to enhance the sustainability, safety, quality, and ecological protection of food. These efforts establish a robust foundation for national food security. The Yangtze River Economic Belt is a key axis for China’s economy. It is home to over 40% of its population and produces 35% of its food. Food security in this region has a direct impact on the national food situation, the quality of life for residents, and long-term stability.

With increasing global climate change [1,2], tightening resource constraints [3,4], and shifting dietary preferences [5,6], food security now extends beyond traditional one-dimensional guarantees. It is a multidimensional system, comprising quantitative, qualitative, ecological, and capacity security. These elements are linked. Together, they determine overall food security levels and trends. Existing studies have advanced the evaluation of food security. However, most focus on provincial-level analysis. Few examine the dynamic evolution and convergence mechanisms of intra-regional differences. Few studies employ a multidimensional framework that integrates production sustainability with qualitative needs from a consumption perspective. The Yangtze River Economic Belt is important both economically and for food security. Its unique position is crucial. The area’s large population and high consumption have a direct impact on the quality of life and stability. Meanwhile, the region’s abundant agricultural resources and significant food production face major challenges.

Based on this, this paper constructs a multidimensional food security evaluation framework with four dimensions: quantity, quality, ecology, and capacity. The study focuses on 131 prefecture-level cities in the Yangtze River Economic Belt from 2009 to 2022. The paper applies the Gini coefficient decomposition to reveal spatial differentiation in food security levels. It characterizes distributional dynamics through kernel density estimation. A convergence model analyzes the spatial and temporal mechanisms of regional differences. This study offers an innovative approach to enhance the theory of multi-scale assessment of food security. It also complements the existing analysis of regional food security, helping the Yangtze River Economic Belt achieve synergistic development of food security and ecological protection.

## 2. Literature review

Food security, vital to the economy and stability, has sparked a wide academic debate. Current research covers:

First, the theoretical connotation of food security. With economic and social development, as well as changes in internal and external environments, the concept of food security has undergone a remarkable dynamic evolution. Early research primarily focused on ensuring the quantitative security of food, emphasizing the increase in production to meet the basic survival needs of the population. In 1943, the United Nations Conference on Food and Agriculture explicitly stated that “a safe, sufficient and adequate supply of food should be the main policy objective of all countries”; this expression laid down the initial policy orientation of food security [7]. With the aggravation of resource and environmental constraints and the upgrading of the population’s consumption structure, the theoretical construction of food security has gradually evolved from a one-dimensional to a multidimensional framework. The 1974 World Food Conference defined food security for the first time in a policy context, defining food security as “the availability of adequate supplies of the world’s basic foodstuffs at any given time, particularly to avoid the event of large crop failures, natural disasters, or other catastrophic situations. Disasters, severe food shortages, and to sustain a steady increase in food consumption in countries with low per capita intakes and to offset fluctuations in production and prices [8]”. In 1982, the Food and Agriculture Organization of the United Nations (FAO) further expanded the concept by proposing that food security should encompass three core objectives: securing an adequate food supply, enhancing the stability of supply flows, and ensuring accessibility for those in need. Accessibility for those in need. Until the World Food Summit in 1996, a “four-dimensional” theoretical framework covering quantity adequacy, quality reliability, access stability, and consumption access was systematically developed, marking an important shift in food security research from a single quantitative dimension to a systematic analytical paradigm [9].

Second, the food security indicator system has developed from unidimensional to multidimensional. Early studies, limited by the availability of theory and data, primarily used indicators such as grain production and stock levels. These were unidimensional and could not reveal heterogeneous features of complex security patterns. As multidimensional frameworks evolved, composite index systems gained mainstream acceptance. Tang and other scholars constructed an evaluation system based on six dimensions: quantity, quality, circulation, economy, ecological resources, and policy, to address new challenges to China’s food security amid international tensions [10]. Gadiso. W.J. et al. developed a household food security index that encompasses availability, access, utilization, and stability, adopting a micro-household perspective. They empirically analyzed regional differences in Ethiopia [11]; Wineman. A’s team explored how food security evolves spatially and temporally by focusing on quantitative, qualitative, and stabilizing dimensions. They assessed the impact of climatic shocks [12]. Saladini, F. et al. integrated the water-energy-food nexus with the Sustainable Development Goals of the Mediterranean region, creating a series of food security indicators. These studies have enhanced the systematization and relevance of food security assessments [13].

Third, research methods for food security. At the methodological level, the single-indicator and comprehensive evaluation methods form a complementary development pattern. The single-indicator method is simple and intuitive, directly assessing regional food security through the food self-sufficiency rate. However, it cannot comprehensively reflect the multidimensional nature of food security. Therefore, the comprehensive evaluation method is more widely used. Xu et al. proposed a weighting technique that combines subjective AHP and objective CRITIC to analyze the spatial and temporal evolution of food security at both global and regional levels [14]. Mathenge, M. et al. GIS technology was used with the small-area analysis method and drew a spatial dimensionality map of food insecurity. They used principal component analysis and a spatial autocorrelation model to provide a basis for subdimensional intervention strategies [15]. Additionally, Po-chi Chen et al. employed data envelopment analysis to evaluate the efficiency of food production. They suggested that food availability should be a top policy priority in low- and middle-income countries, as well as in sub-Saharan Africa, where shortages are most prevalent [16].

Fourth, the current real-world challenges to food security. On the production side, Krishnamurthy, PX. et al. conducted a study on the frequency of extreme weather events (such as floods, droughts, and high temperatures) triggered by climate change, which severely impacts the stability of food production [17]; ecological degradation (soil erosion, soil sanding, water pollution, etc.) continues to erode the quality of arable land, weakening the potential for food production [3]; the process of industrialization and urbanization has led to the intensification of the intensification of non-agriculturalization of arable land, the reduction of planting area at the same time triggered the loss of agricultural labor, affecting the efficiency of the production scale [18]; in the circulation and consumption side, the rise of international trade protectionism, the international price of food fluctuations, increasing the uncertainty and cost pressure of China’s food imports [19].

Fifth, research on the impact of food security issues. A series of problems caused by food security has also received extensive attention from scholars. At the economic level, price fluctuations caused by the imbalance between food supply and demand will push up the level of inflation, increase the cost of living of the population, and threaten the macroeconomic stability of [20]; at the social level, Sassi, M. et al. have found that food shortages in impoverished areas may exacerbate the problem of malnutrition and lead to a vicious circle of “food insecurity and qualitative insecurity”, and even induce social conflicts [21]; at the ecological level, over-cultivation and abuse of fertilizers and pesticides to cope with the pressure on production may lead to the destruction of ecosystems, forming a two-way negative feedback between ecological security and food security [22]; at the strategic level, insufficient food security resilience will weaken the country’s autonomy and voice in global governance, posing a potential threat to national security [23].

Scholars at home and abroad have conducted extensive research on the connotation of food security, the development of the indicator system, and the challenges in reality, which has laid a solid foundation for subsequent research. However, there are still two areas that need to be expanded: first, most of the research on the Yangtze River Economic Zone focuses on the provincial macro scale, lacking in-depth excavation of the heterogeneous characteristics and dynamic evolution mechanism at the prefecture and municipal levels, making it difficult to accurately identify differentiated security risks within the region; and second, the evaluation indexes and intrinsic composition of multidimensional food security need to be deepened. The existing literature often evaluates food security by constructing indicators from the perspective of quantitative security, but it is urgent to conduct in-depth research on how to integrate quantitative, qualitative, ecological, and capacity factors to construct a multidimensional food security indicator system that meets China’s situation, accurately measures China’s multidimensional food security level, and recognizes the structural characteristics and power sources of the dynamic evolution of multidimensional food security. This paper provides theoretical support for the differentiated governance of food security in the Yangtze River Economic Zone by constructing a four-dimensional analytical framework of “quantity-quality-ecology-capacity” and combining it with Dagum’s Gini coefficient decomposition and convergence model. The marginal contribution of this paper is mainly reflected in the following two aspects: at the research perspective level, taking the prefecture-level cities in the Yangtze River Economic Belt basin as the research unit, the spatial heterogeneity characteristics of the high-value cities of food security such as Suzhou, Chongqing and some low-value cities in the upper reaches of the Yangtze River Economic Belt basin are accurately identified through the data refined to the city level, which makes up for the limitation of macro-scale research on the interpretation of the “micro-differences within the region”. At the level of research methodology, Dagum’s Gini coefficient decomposition, kernel density estimation, convergence model, and other methods are comprehensively applied to form a complete analytical chain of “difference measurement-distribution portrayal-convergence mechanism”, which complements the existing literature.

## 3. Description of the research methodology and data

In this paper, we will use the prefecture-level cities from 11 provinces in the Yangtze River Economic Belt as the research objects to construct a multidimensional food security evaluation system, examining four aspects: quantitative, qualitative, ecological, and capacity security. Firstly, the entropy method is used to assign weights and calculate the multidimensional food security index; secondly, the Dagum Gini coefficient and its decomposition method are used to analyze the regional differences in the distribution of the food security level in the Yangtze River Economic Belt; next, the kernel density is used to estimate the dynamics of the distribution of its multidimensional food security; and finally, the convergence analysis is carried out to explore the convergence characteristics of the food security level in different regions.

### 3.1. Construction of multidimensional food security level evaluation indicators

Against the dual backdrop of intensifying global food security challenges and deepening China’s regional coordinated development strategy, it is challenging to comprehensively portray the complex connotation of food security using the traditional unidimensional evaluation system centered on production. Based on this, this paper focuses on the strategic positioning of the Yangtze River Economic Belt as a national grain producing area and an ecological and economic corridor, and constructs a multidimensional evaluation system that includes quantitative, qualitative, ecological, and capacity security, in accordance with the four-dimensional framework of food security of the Food and Agriculture Organization of the United Nations (FAO), and in combination with China’s policy practice of “Hiding food on the land, hiding food in the technology”. Multidimensional evaluation system (see Table 1 for details). From a theoretical level, food security not only requires a stable and sufficient quantitative supply of food, but also needs to consider the rationality of the qualitative structure, the ecological sustainability of the production process, and the balance of regional support capacity. The system breaks through the traditional single production orientation and reveals the systematic characteristics of food security through a four-dimensional synergistic framework: quantitative security guarantees the supply base, qualitative security optimizes the consumption structure, ecological security constrains resource depletion, and capacity security strengthens the development resilience. The four form a closed-loop logic of “production-consumption-environment-support”, reflecting the dynamic coupling relationship of food security at different spatial and temporal scales. It is worth noting that the indicator system employs a two-way evaluation criterion, where positive indicators have a synergistic effect on the level of food security, while negative indicators have a significant inhibitory effect.

**Table 1:** Multidimensional food security indicator system.

Dimension	Indicator	Indicator unit	Indicator characteristics	weights
Quantitative security	grain production	10000 tons	+	20.78%
	food production per capita	kg/person	+	4.46%
	gross output value of agriculture, forestry, livestock, and fisheries	billions	+	7.56%
Qualitative security	pesticide use	ton	–	0.17%
	agricultural diesel use	ton	–	0.46%
	total meat production	10000 tons	+	9.82%
Ecological security	effective irrigated area	1000 Ha	+	7.73%
	fertilizer application rate	10000 tons	–	0.51%
	agricultural plastic film use	ton	–	0.07%
Competence security	rural electricity consumption	kilowatt-hours	+	35.13%
	gross power of agricultural machinery	10000 KW	+	6.93%
	rural disposable income per capita	yuan	+	6.38%

Quantity security is the foundation of food security, which directly reflects the adequacy of food supply. In this paper, we refer to Tang et al. who set the following indicators to measure the quantity security [10], food production is the core indicator to measure the food supply capacity, and its level directly affects the regional food self-sufficiency rate and the degree of external dependence; the per capita production of food can more intuitively reflect the per capita level of food supply, which provides an important basis for the assessment of regional food security; the total output value of agriculture, forestry, animal husbandry and fishery not only covers food production, but also includes the economic value of other agricultural products, which can comprehensively reflect the regional agricultural economic strength and food security economic foundation.

Qualitative security, focused on food quality and structural rationality, is a crucial aspect of food security related to consumption and nutrition. Referring to the research results of Will J, we will incorporate the two negative indicators of pesticide use and agricultural diesel fuel use [24], the former reflecting the potential threat of the production process to the quality of food, and the latter indirectly reflecting the environmental risk brought about by the energy consumption of mechanization; and the total meat production as a positive indicator reflecting the level of development of animal husbandry and the ability of the population to supply protein-based food, which is an important aspect of the measurement of the qualitative security of the region.

Ecological security is a guarantee for the sustainable development of food security [25]. Effective irrigation area reflects the degree of perfection of agricultural irrigation facilities and water use efficiency, and sufficient irrigation water is an important condition to guarantee stable and high food production; chemical fertilizers and the use of agricultural plastic film a negative indicators, respectively, reflecting the risk of soil pollution and white pollution pressure, highlighting the environmental costs of agricultural production.

Capacity security reflects the regional agricultural development capacity and farmers’ living standards, and plays a crucial role in supporting food security. Rural electricity consumption reflects the level of energy consumption for production and living in rural areas, which is an important indicator of the degree of rural economic development and agricultural modernization; the total power of agricultural machinery reflects the level of mechanization of agricultural production, and the higher the degree of mechanization, the more guaranteed the efficiency of agricultural production and food production; the per capita disposable income in rural areas directly reflects the economic situation and the quality of life of farmers, and a higher level of income helps to increase the motivation and capacity of agricultural inputs, which is important for guaranteeing food security.

### 3.2 Multidimensional safety level measurement based on the entropy value method

To objectively quantify the multidimensional characteristics of food security in the Yangtze River Economic Belt, this study employs the information entropy theory for indicator assignment. This method objectively determines the indicator weights based on the principle of information entropy, effectively eliminating the interference of subjective factors in weight allocation. The specific steps are as follows:

(1) Data were standardized.

The following formulas were used to calculate the standardized values, respectively:

The formula for normalizing positive indicators is,


$$r_{ij}=\frac{a_{ij}-\text{min}\;\left(a_{ij}\right)}{\text{max}\;\left(a_{ij}\right)-\text{min}\;\left(a_{ij}\right)}$$ (1)

The formula for normalizing negative indicators is,


$$r_{ij}=\frac{\text{max}\;(a_{ij}\;)-a_{ij}}{\text{max}\;\left(a_{ij}\right)-\text{min}\;\left(a_{ij}\right)}$$ (2)

Where $a_{ij}$ denotes the original value of the j indicator of the i sample, and $r_{ij}$∈[0,1] is the standardized value.

(2) Calculate the weight of each evaluation indicator.

$$P_{ij}=\frac{r_{ij}}{\sum_{i=\text{1}}^{n}r_{ij}}$$ (3)

Where n is the total number of samples.

(3) Information entropy and utility value measurement.

$${\text{E}}_{\text{j}}=-\text{k}\sum {\mathrm{\backslash nolimits}}_{\text{i}=\text{1}}^{\text{n}}{\text{p}}_{\text{ij}}\;\text{ln}\;{\text{p}}_{\text{ij}}$$ (4)

Where i = 1,2,…, n, $\text{k}=\frac{\text{1}}{\text{ln}\;\text{n}}$

The utility value of information entropy $G_{j}$ is further defined to characterize the relative importance of the indicator, and the negative entropy value effect is eliminated by normalization:


$$G_{j}=\text{1}-E_{j}$$ (5)

(4) Calculation of indicator weights.

$$W_{j}=\frac{G_{j}}{\sum_{j=\text{1}}^{m}G_{j}}$$ (6)

(5) Calculate the combined food security score.

$$S_{i}=\sum {\mathrm{\backslash nolimits}}_{j=\text{1}}^{m}W_{j}r_{ij}$$ (7)

Where m denotes the number of indicators in the food security evaluation system, $r_{ij}$ denotes the standardized value of the j indicator in year i, and $a_{ij}$ denotes the raw value of the j indicator in year i.

### 3.3. Gini coefficient and decomposition

Dagum’s Gini coefficient breaks through the limitations of the traditional Gini coefficient in measuring intergroup differences and provides a powerful tool for analyzing the spatial and temporal evolutionary dynamics of the multidimensional food security level differences in the Yangtze River Economic Belt by breaking the overall differences into three parts: intragroup differences, intergroup differences, and hypervariable density differences.

Based on the significant differences in geographic location, resource endowment, economic gradient and policy orientation, this paper divides the Yangtze River Economic Belt into three major subgroups: the downstream region (covering Shanghai, Jiangsu and Zhejiang) is characterized by plains and river network topography, with rapid industrialization and urbanization, and a high degree of modernization of agriculture despite the constraints on arable land resources for food production; the middle reaches (including Anhui, Jiangxi, Hubei and Hunan), as an important commercial grain base in China, are dominated by plains and hills, with a strong dependence on agriculture, but there is still much room for improvement in terms of economic inputs and technological equipment; the upstream area (including Chongqing, Sichuan, Yunnan and Guizhou) is dominated by mountainous plateaus, with outstanding ecosystem vulnerability and the policy mission of “grasping the great protection together”. There is a significant trade-off between food production and ecological protection. This regional division not only aligns with the objective gradient law of regional development, but also highly coincides with the unique methodological advantage of the Dagum model in addressing the differences between discontinuous subgroups.

Based on the above regional division, this paper employs the Dagum Gini coefficient to measure regional differences in multidimensional food security levels within the Yangtze River Economic Zone. The specific calculation steps are as follows:

First, macroeconomic data of each region, including GDP, per capita income, food production, etc., are systematically collected; then, groups are grouped according to the classification of downstream, midstream, and upstream; subsequently, intra-group disparity, inter-group disparity, and hyper-variable density disparity are computed with the help of the formula of Dagum’s Gini Coefficient, respectively. The overall formula of Dagum’s Gini Coefficient is:


$$\begin{array}{c} G=\frac{\sum {\mathrm{\backslash nolimits}}_{j=\text{1}}^{k}\sum {\mathrm{\backslash nolimits}}_{h=\text{1}}^{k}\sum {\mathrm{\backslash nolimits}}_{i=\text{1}}^{n_{j}}\sum {\mathrm{\backslash nolimits}}_{r=\text{1}}^{n_{h}}\left|y_{ij}-y_{hr}\right|}{\text{2}n^{\text{2}}\overset{―}{y}} \end{array}$$ (8)

In this equation, G denotes the overall Gini coefficient, k denotes the number of regions, n denotes the number of cities, $y_{ij}\;\;(y_{hr})$ denotes the multidimensional food security level of the cities in the j(h) region, and $\overset{―}{y}$ denotes the average of the multidimensional food security level of all cities in the Yangtze River Economic Zone.

According to Dagum’s decomposition of the Gini coefficient, the overall Gini coefficient G is systematically decomposed into three components: intra-regional variance, inter-regional variance, and hypervariable density variance. Specifically, intra-regional variance reflects the differential contribution of the multidimensional food security indexes of cities within the three subgroups of the Yangtze River Economic Belt: upstream, midstream, and downstream; inter-regional variance quantifies the differential contribution of the three subgroups; and hypervariance density variance captures the contribution of the phenomenon of cross-overlap between samples to the overall variance. In this paper, hypervariable density variance is characterized by the presence of low-level cities within regions with high levels of food security and the presence of high-level cities within regions with low levels of food security, a phenomenon that exacerbates the overall variance.

### 3.4. Kernel density estimation

Kernel density estimation, as a nonparametric estimation method, can smoothly fit the probability density function of the data, thus effectively revealing the distributional characteristics of the data. In analyzing the distribution dynamics of the multidimensional food security level in the Yangtze River Economic Zone, this paper employs the kernel density estimation method, as calculated by Equations (9) and (10).

Equation (9) represents the general form of kernel density estimation:


$$f(x)=\left(\frac{\text{1}}{Nh}\right)\sum {\mathrm{\backslash nolimits}}_{i=\text{1}}^{N}K\left(\frac{X_{i}-x}{h}\right)$$ (9)

Equation (10) then defines a Gaussian kernel function of the form:


$$K(x)=\frac{\text{1}}{\sqrt{\text{2}\pi }}e^{-\frac{x^{\text{2}}}{\text{2}}}$$ (10)

Where f(x) is the estimated probability density function, N represents the number of observations, i.e., the total number of cities involved in the Yangtze River Economic Belt; $X_{i}$ represents the independently and identically distributed observations, which correspond to the composite scores of each city’s multidimensional food security level; and K(x) is the kernel function, x is the mean value, and h is the kernel width.

### 3.5. Convergence model

Using the α-convergence and β-convergence models, this paper aims to accurately investigate the evolution of regional differences in multidimensional food security levels in the Yangtze River Economic Zone, i.e., to clarify whether the differences tend to converge or diverge over time, and at the same time, to analyze whether the regions with lower levels of food security have the ability to grow at a faster rate in order to catch up with the high level regions.

#### 3.5.1. α-convergence.

The α-convergence reflects the dynamic trend of the differences in the food security levels between different regions. By calculating the coefficient of variation of the level of food security, it is possible to effectively determine whether there is a convergence feature in the level of food security. If the coefficient of variation shows a decreasing trend from year to year, it indicates that the difference in food security level between regions is gradually narrowing, i.e., there is α convergence. In this study, the coefficient of variation is utilized to portray the convergence of α. The specific formula is as follows:


$$\begin{array}{c} \sigma =\sqrt{\frac{\sum {\mathrm{\backslash nolimits}}_{i=\text{1}}^{n}{\left(D_{i,t}-\overset{―}{D_{i,t}}\right)}^{\text{2}}}{n}}/\overset{―}{D_{i,t}} \end{array}$$ (11)

Where i denotes different cities in the Yangtze River Economic Belt, and t denotes the year, and $D_{i,t}$ is used to denote the multidimensional food security index of city i in year t.

#### 3.5.2. β-convergence.

The theory of β-convergence examines whether regions with lower levels of multidimensional food security can catch up with regions with higher levels of food security at a faster pace, thereby gradually reducing regional disparities and ultimately achieving convergent growth. According to the heterogeneity of the convergence path, this study subdivided β-convergence into two types of models: absolute β-convergence and conditional β-convergence.

Absolute β-convergence assumes that all regions will eventually converge to an identical steady-state level, regardless of their initial level of food security. Conditional β-convergence, on the other hand, analyzes whether the food security levels of regions converge to similar steady-state levels, controlling for other influences. This implies that although there are differences in food security levels across regions, these differences will diminish after controlling for other variables.

The model for absolute beta convergence is shown below:


$$\begin{array}{c} \text{ln}\left(\frac{D_{i,t+\text{1}}}{D_{i,t}}\right)=\alpha +\beta \;\text{ln}\;D_{i,t}+\mu_{i}+\nu_{t}+\varepsilon_{it} \end{array}$$ (12)

Denote the multidimensional food security of city i in year t by $D_{i,t}$, the multidimensional food security of city i in year t+1 by $D_{i,t+\text{1}}$, and the spatial fixed effects, time fixed effects and randomized disturbance terms by $\;\mu_{i}$, $\nu_{t}$ and $\varepsilon_{it}$, respectively.

The β-convergence of the multidimensional food security level is affected by many factors. In order to improve the accuracy of the prediction of the convergence pattern of the multidimensional food security level, this paper adds other control variables on the basis of the absolute β-convergence model, so as to get the conditional β-convergence of the econometric model as follows:


$$\begin{array}{c} \text{ln}\left(\frac{D_{i,t+\text{1}}}{D_{i,t}}\right)=\alpha +\beta \;\text{ln}\;D_{i,t}+\lambda \sum {\mathrm{\backslash nolimits}}_{j=\text{1}}^{n}{Control}_{i,t}+\mu_{i}+\nu_{t}+\varepsilon_{it} \end{array}$$ (13)

Where α is the constant term, β is the convergence coefficient. If β is significantly less than 0, it indicates that there is a convergence feature in the level of multidimensional food security, and vice versa, there is a dispersion feature, and the speed of convergence can be calculated by −ln(1+β)/T. λ denotes the coefficient of control variables, and ${Control}_{i,t}$ denotes the control variables affecting the degree of multidimensional food security, which mainly include urbanization rate, financial support, economic development level, and high industrial structure.

Among them, the urbanization rate reflects the spatial agglomeration of the population, and its changes will lead to adjustments in the allocation of arable land resources and the structure of food consumption, thus affecting the pattern of regional food security, which is measured by the ratio of the urban population to the total population; the strength of financial support reflects the strength of the government’s support for agriculture and food security, and higher financial support usually helps to enhance the production capacity of food and guarantee food security, which is measured by the ratio of general budget expenditure to regional GDP; the level of economic development is measured by GDP per capita, which reflects the overall level of regional economic development; the heightened industrial structure is calculated by the following formula. The level of economic development is measured by GDP per capita, reflecting the overall level of regional economic development; the heightened industrial structure is calculated by the following formula: heightened industrial structure = 1 × primary industry value added ratio + 2 × secondary industry value added ratio + 3 × tertiary industry value added ratio Which directly reflects the degree of optimization and upgrading in industrial structure as it evolves from low-value-added to high-value-added sectors. The more advanced the industrial structure is, the higher the resource allocation efficiency is, and the more significant the positive empowering effect on food security is. For example, the development of the service industry can improve the efficiency of food circulation.

### 3.6. Data sources

Considering the authenticity and availability of the data, this paper selects the panel data of a total of 131 prefecture-level administrative units (including prefecture-level cities and autonomous prefectures) in 11 provinces and cities (including 2 municipalities directly under the central government and 9 provinces) in the Yangtze River Economic Belt from 2009 to 2022, which are mainly derived from the China Statistical Yearbook, the China Regional Statistical Yearbook, the China Urban Statistical Yearbook, as well as the data officially released by the statistical bureaus of some prefectural cities. The missing values of individual years are filled in by interpolation.

## 4. Empirical analysis

### 4.1. Measurement and analysis of multidimensional food security levels

Based on the entropy value method, this paper systematically measures the multidimensional food security level of 131 cities in the Yangtze River Economic Zone from 2009 to 2022. By constructing an evaluation system with four dimensions, namely, quantitative security, qualitative security, ecological security and capacity security, the results are shown in Table 1, and the weight distribution structure of each dimension shows significant differences: quantitative security (32.80%) and capacity security (48.44%) constitute the core support of the system, with a combined contribution rate of 81.24%; qualitative security (10.45%) and ecological security (8.31%) embody the auxiliary security function. Qualitative security (10.45%) and ecological security (8.31%) reflect the auxiliary security function. To reveal the spatial and temporal heterogeneity of regional food security, the study follows the logic of “whole-local-individual” and measures it at three levels: the whole sample, sub-regions, and sub-cities, focusing on the temporal evolution of multidimensional food security, spatial differentiation, and differentiation at the city level.

#### 4.1.1. Full Sample Measurement Results.

**Fig 1:**
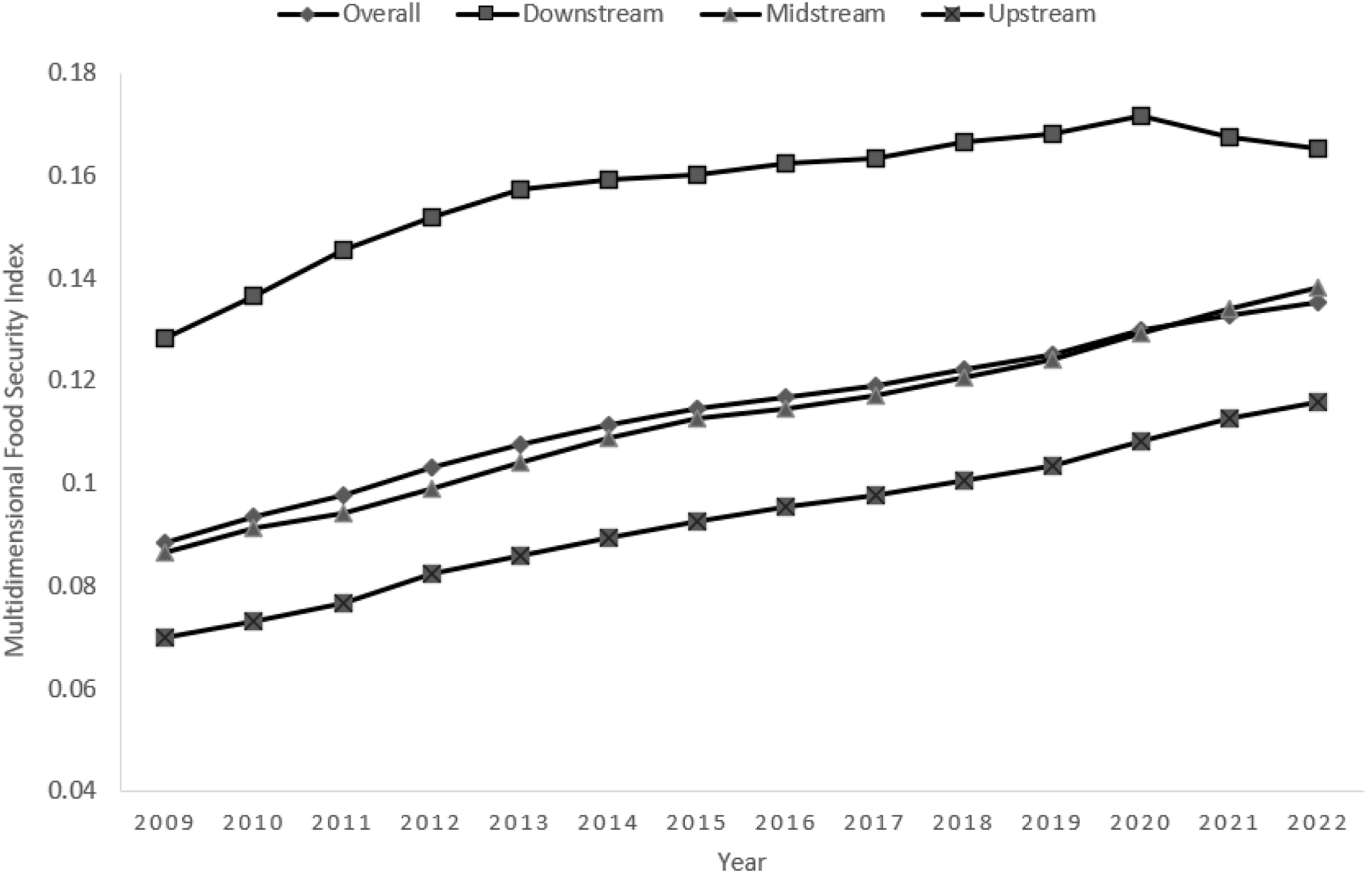
Trends in Multidimensional Food Security Levels in the Yangtze River Economic Zone, 2009-2022.

According to Fig 1, the overall food security level of the Yangtze River Economic Belt has shown a steady upward trend from 2009 to 2022, with the food security index increasing from 0.089 in 2009 to 0.135 in 2022, representing an average annual growth rate of approximately 3.3%. This trend suggests that the food security capacity of the Yangtze River Economic Belt has improved significantly over the past decade or so.

Furthermore, the multidimensional food security index for each city from 2009 to 2022 is calculated as an annual average and ranked. The results show that the top five cities are Suzhou, Chongqing, Wuxi, Yancheng, and Xuzhou, in that order. The cities at the bottom of the rankings are mostly located upstream. This distributional feature echoes the trend in Fig 1, which shows that food security levels in the lower reaches are significantly higher than those in the upper reaches, further corroborating the pattern of interregional differences in food security levels at the city level. These differences reflect the economic and technological superiority of the downstream regions in the dimension of capacity security (e.g., agricultural mechanization, capital investment), but they also highlight the objective reality of the upstream regions, which are subject to geographical conditions and ecological constraints.

#### 4.1.2. Subregional measurement results.

From the results of the subregional measurements in Fig 1, the multidimensional food security levels in the upper, middle, and lower reaches of the Yangtze River Economic Belt show a pattern of “significant gradient differences”.

Specifically, the food security index in the upstream region increased from 0.07 in 2009 to 0.12 in 2022, despite a low starting point and the arduous task of ecological protection. It may be constrained by the mountainous plateau topography and ecological protection policies, as well as a relatively weak agricultural resource endowment, and certain trade-offs between food production and ecological protection. In addition, the policy direction emphasizes the priority of ecological protection, which also restricts the development of agricultural production to a certain extent; the middle reaches of the region, as a nationally important commodity grain production base, food security index that has grown steadily from 0.09 in 2009 to 0.14 in 2022, showing a stable and positive development trend. Relying on the topography of plains and hills, the region has traditional advantages in terms of quantitative security. Through gradually increasing agricultural economic inputs and promoting the upgrading of technology and equipment, the dimension of capacity security has been continuously strengthened. The transition from “traditional food production” to “high-quality security” has been achieved, and it has become an important guarantee for food security in the Yangtze River Economic Zone; the downstream area, as a highland for economic development, is dominated by the plains and river networks, with a high level of industrialization and urbanization and a prominent degree of modernization of agriculture, and at the same time, a perfect agricultural industry chain and advanced management experience have further strengthened its food security guarantee capability. Multi-dimensional food security level has always maintained a leading edge from 2009 to 2022, with the index steadily rising from 0.13 to 0.17, and the high-value area of food supply is mainly concentrated in the central and northern regions of Jiangsu.

### 4.2. Regional differences in multidimensional food security levels and decomposition

Based on the multidimensional food security level measurement above, it is found that there are significant regional differences in the food security level of the Yangtze River Economic Belt. In this section, the Dagum Gini coefficient decomposition method will be applied to analyze the multidimensional food security levels in the upper, middle, and lower reaches of the Yangtze River Economic Belt, examining overall differences, intra-regional differences, inter-regional differences, and the contribution of various sources of these differences.

#### 4.2.1. Overall differences.

**Fig 2:**
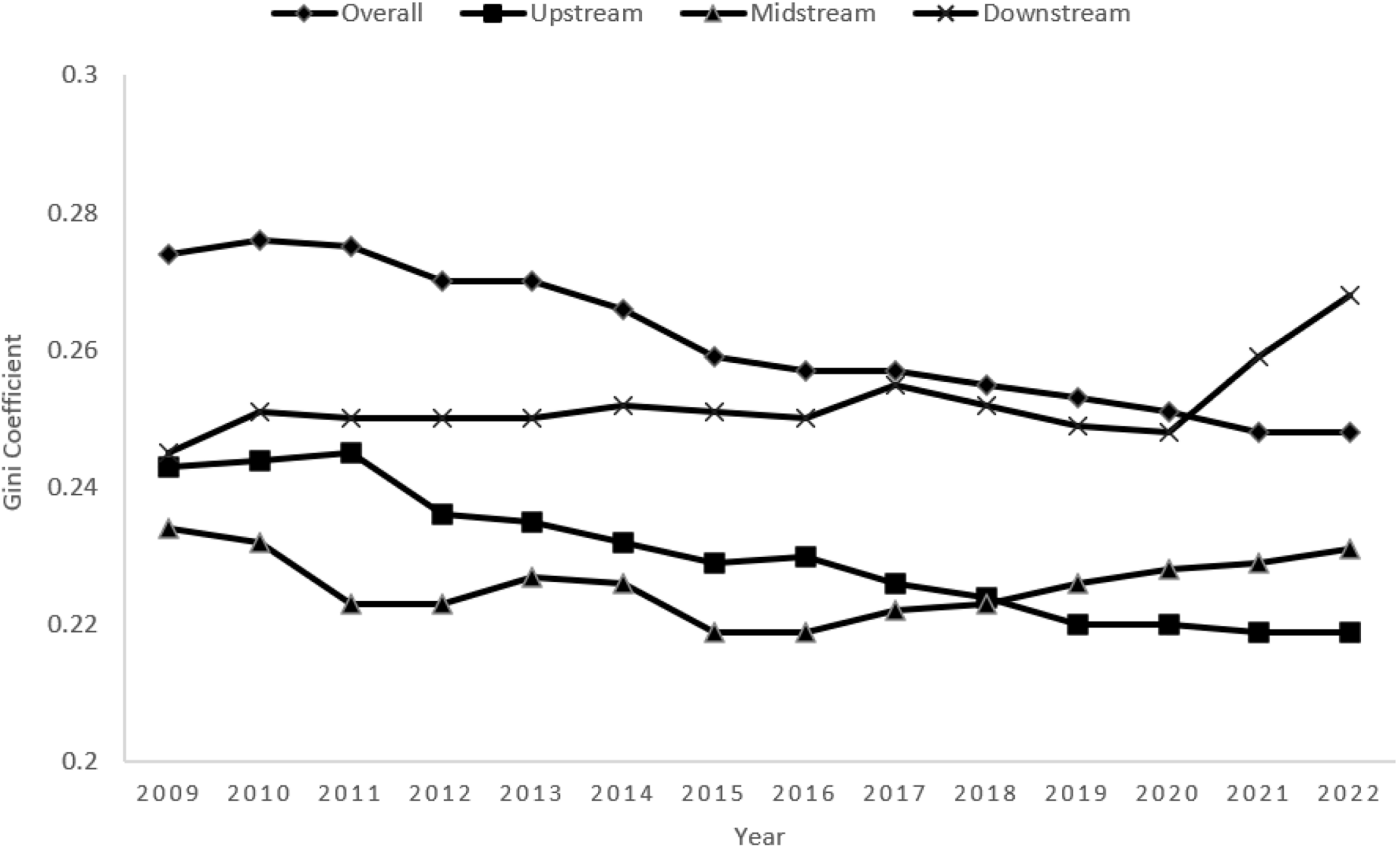
Overall differences in multidimensional food security levels in the Yangtze River Economic Zone and trends in intra-regional differences.

According to the trend of the Gini coefficient shown in Fig 2, the overall difference in multidimensional food security levels in the Yangtze River Economic Zone shows a continuous, fluctuating downward trend between 2009 and 2022. Specifically, the Gini coefficient fluctuates from 0.274 in 2009 to 0.248 in 2022, with a cumulative decrease of 9.5 per cent, which indicates that the degree of dispersion of food security levels between regions continues to decrease, reflecting the gradual enhancement of regional coordination in the area of food security in the Yangtze River Economic Belt, and that policy interventions and the optimization of resource allocation have played a positive role in promoting balanced regional development.

In terms of stage-by-stage characteristics, the Gini coefficient declined markedly from 0.274 to 0.259 during the period 2009–2015, with an average annual decline of approximately 0.25%. The change in this stage is attributed to the continued promotion of national policies to strengthen and benefit agriculture, including initiatives such as increasing investment in agricultural infrastructure construction, promoting modern agricultural technology, and improving the food subsidy mechanism to enhance farmers’ incentives to grow food, effectively bridging the gap in the level of food security between regions. During the 2016–2022 period, the decline in the Gini coefficient slowed, from 0.257 to 0.248, primarily due to deep-rooted structural contradictions, including differences in industrial structure and uneven resource endowments between regions. Although the coordinated development of the region has achieved certain results, some regions are constrained by factors such as the arduous task of ecological protection and the lagging level of economic development, which have limited the further narrowing of regional differences. Overall, although the overall differences in the level of multidimensional food security in the Yangtze River Economic Belt have narrowed, the problem of interregional imbalance still exists.

#### 4.2.2. Intra-regional differences.

Table 2 reports the intra-regional Dagum Gini coefficient calculations of multidimensional food security levels in the upstream, midstream, and downstream regions of the Yangtze River Economic Belt From 2009–2022, the average annual Gini coefficients of the upstream, midstream, and downstream regions are 0.230, 0.226, and 0.252, respectively, indicating that the downstream region has the largest internal differences and the midstream region has the smallest internal differences. This phenomenon is mainly due to the downstream region of Shanghai, Suzhou and other core cities by virtue of advanced grain reserve facilities, supply chain digitalization and other ability to form a polarization effect of security dimension advantages, while some marginal cities by the impact of non-agriculturalization of arable land, the lack of investment in food production resources, resulting in the development of the region within the imbalance; the middle reaches of the region to the main grain producing areas such as the Jianghan Plain and the Dongting Lake Plain as the main body, agricultural production conditions. The middle reaches of the region are dominated by grain-producing areas such as the Jianghan Plain and the Dongting Lake Plain, with similar agricultural production conditions, relatively small differences in the mechanization rate and the level of modernization of warehousing, and a good basis for synergistic development within the region, thus resulting in smaller internal differences.

**Table 2:** Statistics on differences in the level of multidimensional food security in the Yangtze River Economic Zone.

regional	annual average value	intra-regional	annual average value
upper reaches of a river	0.230	Top-Middle	0.252
the middle stretches of a river	0.226	Up-Down	0.333
lower reaches of a river	0.252	Middle-Lower	0.274

Furthermore, the evolution of regional differences also exhibits stage characteristics, with the Gini coefficient in the upstream region fluctuating from 0.24 in 2009 to 0.22 in 2022, which may be attributed to the implementation of the policy of returning farmland to forests after 2016. Through the ecological compensation mechanism, the policy effectively reduces the development gap between mountainous areas and river valleys, and promotes balanced development within the region; the Gini coefficient in the middle reaches shows a fluctuating downward and then upward trend, and this change may be related to the increase in the construction of agricultural infrastructure in the middle reaches of the region in recent years, and the initial stage of investment has led to the faster development of some areas, while the later stage, with the gradual improvement of infrastructure, the difference between regions has widened; the downstream region is affected by the accelerated non-agriculturalization of arable land, and the Gini coefficient climbs from 0.25 to 0.27 from 2012 to 2020, a phenomenon that shows that despite the overall food security level in the downstream region, the accelerated process of urbanization and the reduction of arable land resources have negatively impacted intra-regional equilibrium. The relatively low level of food security in some cities due to the high degree of non-agriculturalization of arable land has led to widening intra-regional disparities.

Overall, the Gini coefficient of the multidimensional food security level in the Yangtze River Economic Belt has decreased from 0.28 to 0.24, with a synchronized contraction of intraregional differences, confirming the effectiveness of the policy of the “coordinated food security governance system”.

#### 4.2.3. Interregional differences.

**Fig 3:**
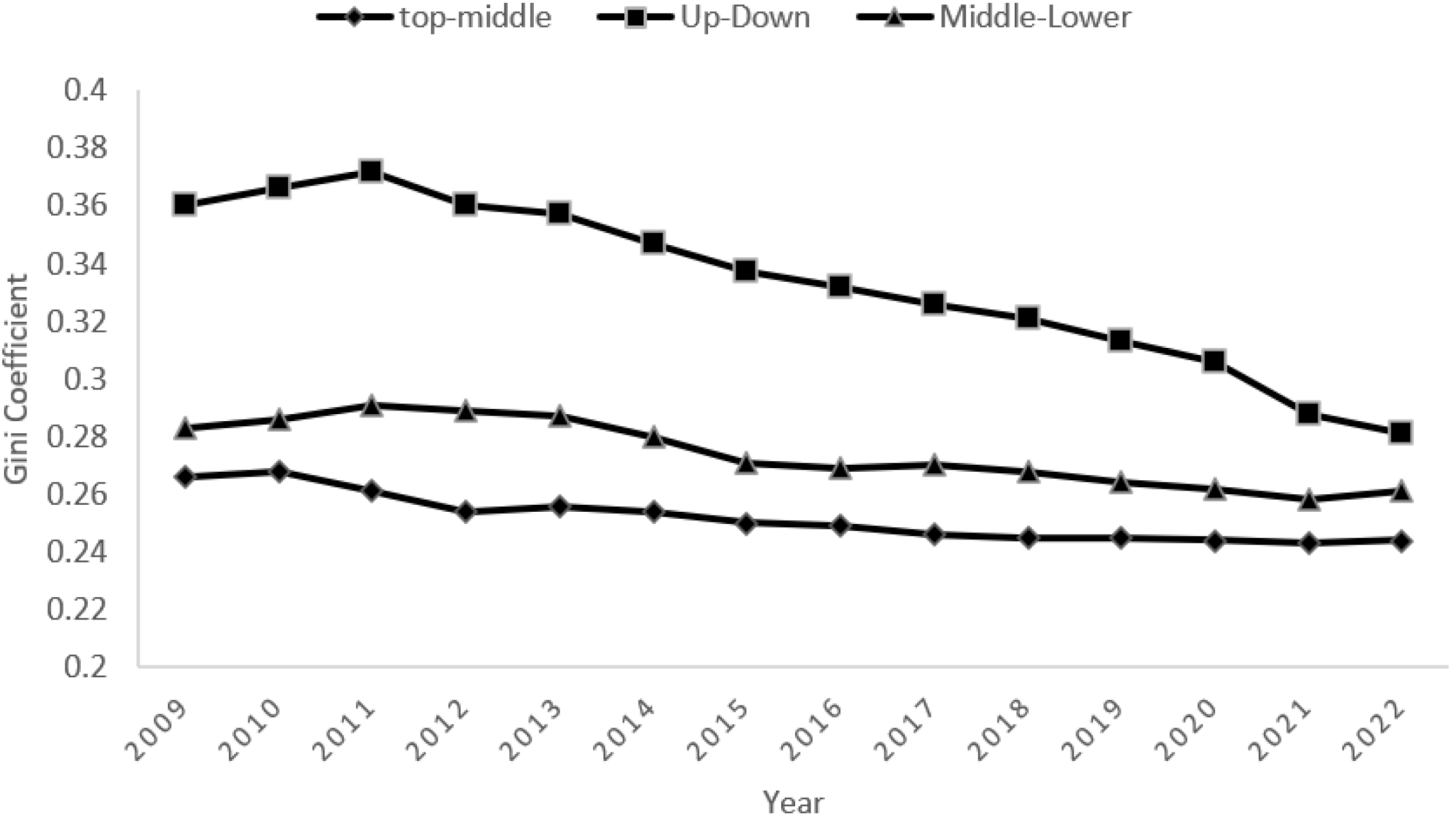
Overall differences in multidimensional food security levels in the Yangtze River Economic Zone and trends in inter-regional differences.

According to Table 2, the overall downward trend in the Gini coefficient between regions of the Yangtze River Economic Belt indicates a gradual narrowing of the differences in food security levels between regions. Specifically, the annual average values of the Gini coefficients between the upper-middle, upper-lower, and middle-lower groups of regions are 0.252, 0.333, and 0.274, respectively. This indicates that the difference between the middle and lower reaches is the largest, and the difference between the upper and middle reaches is the smallest (Fig 3).

According to the time trend in Fig 3, the Gini coefficient between the upper and lower regions fluctuated from 0.36 in 2009 to 0.281 in 2022, showing that the difference between the upstream and downstream regions has continued to ease over the study period, and that coordinated development in the region has achieved some success. The Gini coefficient between the upper and middle regions fluctuates from 0.266 in 2009 to 0.244 in 2022, with an overall narrowing trend, indicating that the gap in food security levels between the two regions is gradually closing. The Gini coefficient between the middle and lower regions decreases from 0.283 in 2009 to 0.261 in 2022, indicating a shrinking difference and reflecting the gradual convergence of the middle and lower regions in food security capacity building. Further analysis reveals that the narrowing of interregional differences is primarily due to policy interventions and the optimal allocation of resources. For example, national investment in agricultural infrastructure construction in the midstream and upstream regions has increased significantly, contributing to improved food security in these areas. Additionally, increased technology sharing and industrial collaboration have contributed to narrowing interregional disparities. However, despite the overall positive trend, the differences between upstream and downstream remain relatively large, indicating that uneven interregional development still exists.

#### 4.2.4. Sources of regional variations and contribution rates.

**Fig 4:**
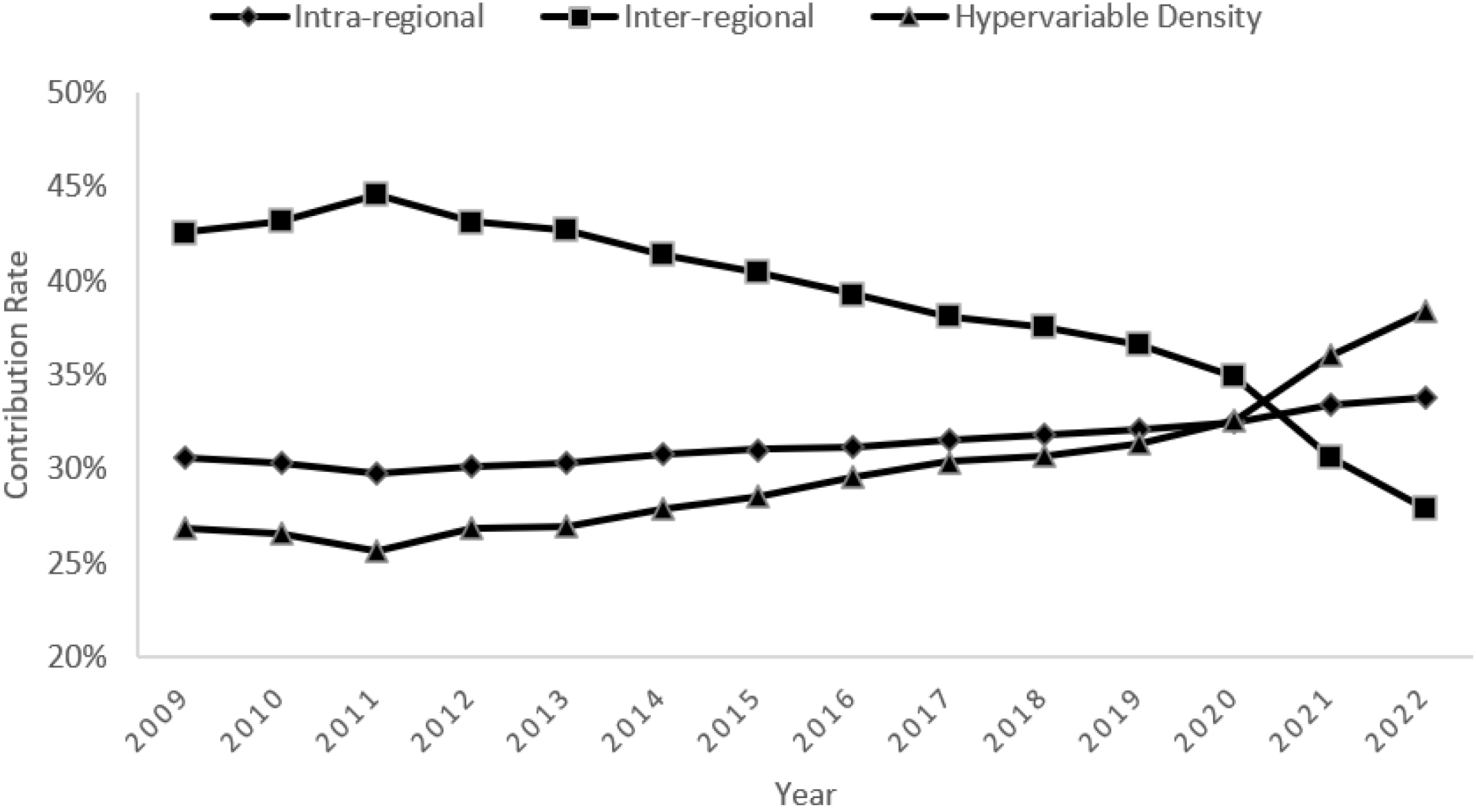
Trends in overall sources of variation in the Yangtze River Economic Zone.

According to Fig 4, the contribution of inter-regional differences to the overall differences is high at the beginning of the study, and then shows a fluctuating downward trend, falling to 28% in 2022, indicating that the influence of the disparity in food security levels between regions on the overall differences is gradually weakening, which may be attributed to the integration policy of the Yangtze River Economic Belt, which promotes the flow of resources across the region and synergistic development, and effectively bridges the development gaps between the upstream, central and downstream regions. The contribution of intra-regional disparities is relatively stable, with a slight increase in the latter part of the period, reflecting the persistent and increasing impact of the imbalance in food security levels among cities in the region on the overall disparities. This may stem from development imbalances within regions, such as the food security levels of some cities in the downstream region being more affected by the defarming of arable land, leading to wider intra-regional disparities.

The contribution of hypervariable density gradually climbs from about 26% to nearly 38% in 2022, highlighting the increasing influence of inter- and intra-regional interactions on overall variation. This may be due to the intertwining of interregional industrial transfers and technology diffusion with the development characteristics of cities within the region, which together shape the complex pattern of food security disparities.

### 4.3. Dynamics of multidimensional food security level distribution

#### 4.3.1. Distribution location.

**Fig 5:**
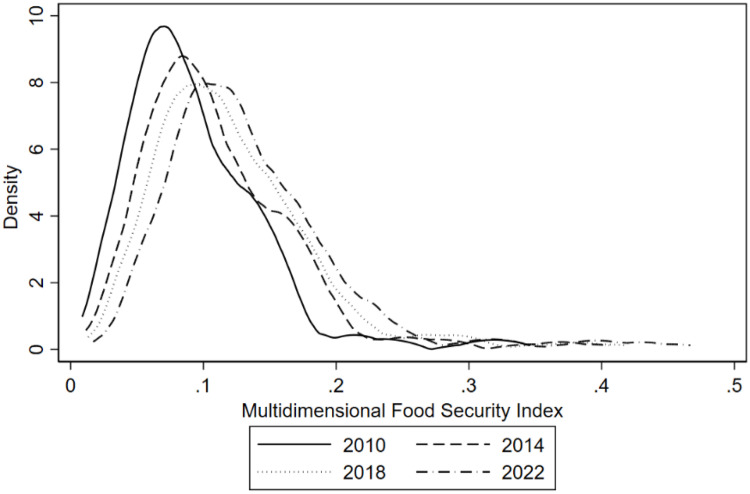
Dynamics of multidimensional food security level distribution in the Yangtze River Economic Zone.

According to the kernel density estimation curve in Fig 5, the high-density regions of the Yangtze River Economic Belt in terms of multidimensional food security levels show significant downstream clustering characteristics. From the peak position, the height of the main peak of the kernel density curve in the downstream region is significantly higher than that in the middle and upper reaches, indicating that the downstream provinces have a higher concentration of resources in the multidimensional food security indicators; the secondary peak position in the middle reaches is relatively low and deviated to the right, implying that there is a spatial “catching-up effect” of food security level in the middle reaches, but due to the lagging of the upgrading of industrial structure and insufficient strength of environmental regulations, a stable and efficient spatial agglomeration has not yet been formed; the upstream region has a high concentration of multidimensional food security level. However, due to the lagging industrial structure upgrading and insufficient environmental regulation, a stable and efficient spatial agglomeration has not yet been formed; the wave peaks in the upstream region show the superposition of multiple peaks, which reflects the heterogeneous distribution of food production efficiency and supply chain stability between agriculture in mountainous areas and agriculture in plains.

#### 4.3.2. Distribution patterns.

In 2010, the nuclear density curve showed obvious right skewness, with a short trailing tail on the left and a prominent long tail on the right, indicating that the proportion of low food security level areas is relatively high, and a few high-security level areas have formed an “island effect”; by 2014, the degree of right skewness weakened and the kurtosis declined slightly, and the density of medium-security level zones was significantly increased in the region. From 2018 to 2022, the curve gradually tends to be symmetrically distributed, and the density decay rate on both sides of the main peak tends to equalize, with the medium-safety level interval becoming the main body, the proportion of low-safety level areas compressed, and the density of high-safety level areas rising but not forming an independent sub-peak. This evolutionary trajectory reflects the formation of an olive-shaped structure of food security in the Yangtze River Economic Zone, with the problem of low-level concentration effectively alleviated in the early stages, and the region as a whole evolving towards a balanced and normalized state of security.

#### 4.3.3. Distributional extensibility.

The thickness of the right tail of the curve is significantly larger than that of the left tail, suggesting a stronger spatial spillover effect in regions with high food security levels. The kernel density curve of the downstream region still exhibits a smooth decay in the interval above 0.8, indicating that its food security system has a stronger extension capacity and may benefit from the technology diffusion and market integration facilitated by the integration of the Yangtze River Delta. In contrast, the right tail of the upstream region has a faster decay rate, and the kernel density value decreases sharply in the range above 0.7, suggesting that the high mountain valley landscape and the lagging transportation infrastructure constrain the gradient jump in food security level.

#### 4.3.4. Polarization phenomena.

From 2010 to 2014, the nuclear density curve always maintained a single-peak pattern, and no independent sub-peak was formed despite the existence of a slight undulation on the right side, indicating that the level of food security in the region has not yet been significantly polarized, and that the gradient difference between high and low level areas is still dominated by a continuous transition; In 2018, a faint secondary peak began to emerge on the right side of the curve. However, its peak height was less than one-third of that of the main peak, and there was a distinct density connection zone between it and the main peak area, indicating that local high safety level clusters were beginning to appear, but they had not yet formed a discontinuous division from the main distribution area; in 2022, the characteristics of the sub-peak were slightly strengthened, but still have not broken through the single-peak dominated pattern, reflecting that the polarization trend of the food security of the Yangtze River Economic Belt is in the nascent stage, and that the “core-periphery” structure within the region has emerged initially, but a stable multi-polar differentiation state has not yet been formed. This suggests that the polarization trend in food security within the Yangtze River Economic Zone is still in its embryonic stage, and the “core-edge” structure in the region is beginning to emerge, although it has not yet formed a stable multipolar state. This feature is in line with the spatial development model of the Yangtze River Economic Belt of “axis linkage and node support”, i.e., the food security advantages of the central cities and urban agglomerations have been gradually emphasized, but they have not yet formed a “siphon effect” or “radiation fault” on the surrounding areas.

### 4.4. Multidimensional food security level convergence analysis

After analyzing the distribution dynamics of multidimensional food security and the decomposition of differences, this paper further explores the evolutionary trend of the differences in the levels of multidimensional food security in China. To achieve this, it utilizes σ-convergence and β-convergence models to analyze the convergence characteristics of the differences between different regions.

#### 4.4.1. α-convergence.

**Fig 6:**
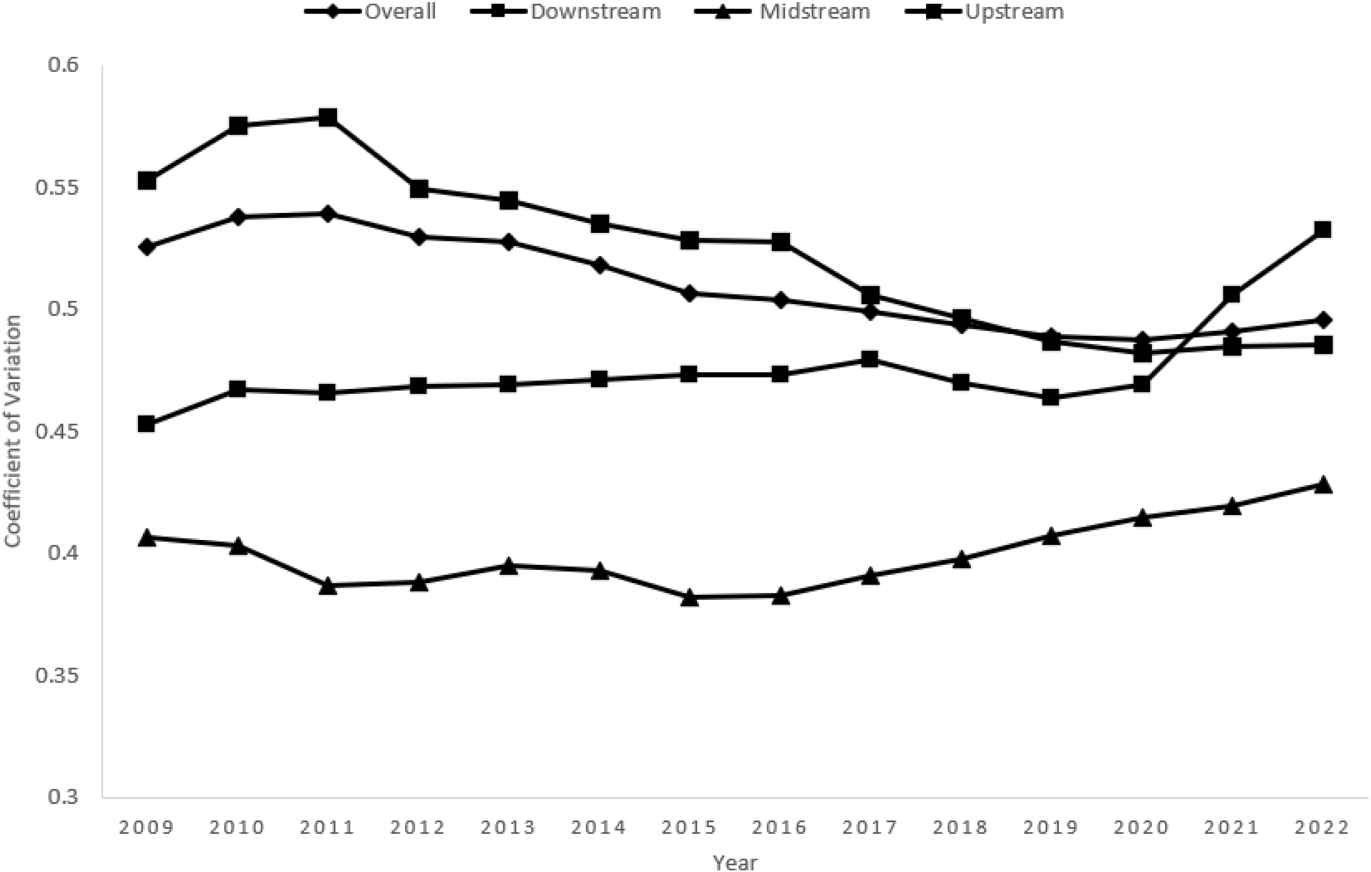
Trend of the coefficient of variation of multidimensional food security level in the Yangtze River Economic Zone.

According to Fig 6, the overall coefficient of variation of the multidimensional food security level in the Yangtze River Economic Belt experienced a small increase from 2009 to 2011, and then entered a declining channel to 0.496 by 2020, indicating that the relative differences in food security levels between regions narrowed during the study period. This trend preliminarily verifies the existence of a certain degree of α-convergence in the multidimensional food security level of the Yangtze River Economic Belt.

Sub-regionally, the coefficient of variation in the upstream region was at a high level in 2009–2011, and showed a significant downward trend after the implementation of *the Outline of the Development Plan for the Yangtze River Economic Belt* in 2016 to 0.485 in 2022, which may be attributed to the fact that the upstream policy of ecological priority has suppressed the expansion of crude agriculture, and provinces and cities have significantly narrowed the differences in the level of food security through the enhancement of the construction of agricultural infrastructure, the acceptance of industrial transfer, and the synergy of ecological protection. This may be due to the fact that the ecological priority policy suppresses the expansion of crude agriculture, and the provinces and municipalities have strengthened the construction of agricultural infrastructure, the transfer of industries, and the synergy of ecological protection, so that the differences in the level of food security are significantly narrowed by the convergence effect. The coefficient of variation in the midstream region as a whole remains relatively stable and low, with small fluctuations. This indicates that the development of food security levels in provinces and cities in the midstream region is more balanced, with stronger industrial homogeneity and collaboration within the region, and smaller regional differences in food production, circulation, and security systems.

The coefficient of variation in the downstream region is relatively stable in the early stage and rises significantly in the late stage, which may be related to the downstream region of the Yangtze River Delta urban agglomeration arable land reduction leading to a decline in food self-sufficiency, coupled with the acceleration of food security in some provinces and cities to promote food security to a higher quality of the transition, the development of high value-added agriculture, and the pace of the transition within the region is inconsistent, resulting in the expansion of the internal differences on the overall convergence of the trend of a certain perturbation.

#### 4.4.2. Absolute beta convergence.

According to Table 3, the multidimensional food security level of the Yangtze River Economic Belt exhibits significant convergence in both the aggregate and regional levels, including the upper, middle, and lower regions. In the full sample estimation, the β coefficient is −0.148 and significant at the 1% level, indicating that the average annual growth rate of the region with a lower initial security level is 14.8% higher than that of the region with a higher initial level, i.e., the less developed region is catching up with the more developed region at a faster rate. Sub-regionally, the absolute value of the β coefficient in the middle reaches is the largest (−0.219), and the convergence speed reaches 1.90% significantly higher than that in the upstream and downstream, which is closely related to the higher efficiency of technological diffusion under the positioning of its main grain producing area and the stronger homogenization of policy implementation; the downstream area has the lowest convergence speed despite the significant negative β coefficient (−0.168), suggesting that the convergence blockage within the YRD urban agglomeration is caused by the crowdedness of non-agricultural resources and the segmentation of the factor market. The downstream area exhibits the lowest convergence speed, despite a significant negative β coefficient (−0.168), indicating that convergence is hindered by non-agricultural resource crowding and factor market segmentation within the YRD urban cluster.

**Table 3:** Results of the absolute β convergence test for the multidimensional food security level in the Yangtze River Economic Zone.

variant	population (statistics)	upper reaches of a river	the middle stretches of a river	lower reaches of a river
β	−0.148***	−0.188***	−0.219***	−0.168***
	(0.0149)	(0.0211)	(0.0203)	(0.0574)
the constant term α	−0.294***	−0.400***	−0.430***	−0.337***
	(0.0319)	(0.0482)	(0.0429)	(0.109)
urban fixed effect	yes	yes	yes	yes
year fixed effects	yes	yes	yes	yes
convergence rate	0.0123	0.0160	0.0190	0.0142
sample size	1,703	611	767	325
R^2^	0.129	0.255	0.198	0.200

Note: *, **, *** indicate significance at the 10%, 5%, and 1% levels, respectively, with standard errors in parentheses.

#### 4.4.3. Conditional β-convergence.

The results of the conditional β-convergence test, based on control variables, show (see Table 4) that the multidimensional food security level of the Yangtze River Economic Belt still exhibits a significant regional convergence feature after the inclusion of control variables such as GDP per capita, urbanization rate, government intervention, and industrial upgrading. The β coefficient of the overall sample is −0.154, which is significantly negative at the 1% level, indicating that the average annual growth rate of the initial low-level region is 15.4% higher than that of the initial high-level region, and the convergence rate is 1.29%, which verifies the robustness of the conditional convergence path. Sub-regionally, the fastest convergence speed is in the middle reaches, with the absolute value of β coefficient reaching −0.223, which is closely related to the homogenization of policy implementation in its main food-producing areas and the high efficiency of technology diffusion; The β coefficient of the downstream region is significantly negative −0.176, but the speed of convergence is the lowest, reflecting the “double-edged sword” effect of urbanization and industrial upgrading in the Yangtze River Delta region, and the significantly positive coefficient of the urbanization rate implies the marginal improvement of food security due to the interconnection of infrastructure in the urban agglomerations, but the fragmentation of arable land due to the expansion of the non-agricultural industry still reduces the power of convergence. Government intervention in the upper reaches significantly inhibits convergence, likely due to ecological function area policies restricting the flexibility of agricultural production factor allocation.

**Table 4:** Results of the absolute β convergence test for the multidimensional food security level in the Yangtze River Economic Zone.

variant	population (statistics)	upper reaches of a river	the middle stretches of a river	lower reaches of a river
β	−0.154***	−0.213***	−0.223***	−0.176***
	(0.016)	(0.023)	(0.021)	(0.057)
ln_gdp_pc	0.002	−0.016	−0.025	0.143*
	(0.019)	(0.020)	(0.030)	(0.075)
urban_ratio	−0.017	−0.024	−0.007	0.144
	(0.019)	(0.016)	(0.036)	(0.107)
gov_intervene	−0.031	−0.059**	0.019	0.027
	(0.022)	(0.023)	(0.080)	(0.071)
industry_upgrade	−0.002	−0.004	0.014	0.004
	(0.004)	(0.004)	(0.015)	(0.010)
the constant term α	−0.329***	−0.476***	−0.486***	−0.715***
	(0.057)	(0.077)	(0.075)	(0.211)
urban fixed effect	yes	yes	yes	yes
year fixed effects	yes	yes	yes	yes
convergence rate	0.0129	0.0184	0.0194	0.0149
sample size	1,703	611	767	325
R^2^	0.131	0.267	0.200	0.226

## 5. Discussion

This paper discusses the theoretical framework, research methodology, and empirical analysis of the multidimensional food security level of the Yangtze River Economic Belt, but there are some shortcomings, and future research can be further supplemented and improved from the following aspects:

(1) Due to data availability, only statistical yearbook data at the prefecture level were used. However, in food security research, more micro-level data (e.g., at the farm household level, plot level, etc.) can provide richer information and more accurate analysis results. Future research can integrate multiple data sources, such as conducting field research to obtain firsthand farm household survey data, and combining satellite remote sensing data to monitor information, including grain planting areas and growth, to complement and validate existing prefecture-level city data.
(2) The methods of Gini coefficient decomposition, kernel density estimation, and convergence modeling applied in this paper provide powerful tools for analyzing the differences in food security levels and the dynamic evolution of food security, but these methods assume, to a certain extent, a linear relationship between the variables. In reality, however, many factors within the food security system may exhibit complex, nonlinear interactions. Future research could incorporate nonlinear analysis methods, such as nonlinear regression models and artificial neural networks, to more effectively capture the complex relationships within the food security system and enhance the fitting accuracy and explanatory power of the models.
(3) The issue of food security involves several disciplinary fields such as economics, agricultural science, ecology, and sociology. This paper primarily conducts research from an economic perspective, and interdisciplinary research can be further strengthened in the future by integrating the theories and methods of multiple disciplines. This will contribute to a more comprehensive and in-depth understanding of food security issues, providing a solid scientific basis for the development of effective solutions.

## 6. Conclusions and policy recommendations

### 6.1. Conclusion

This paper draws the following conclusions from an in-depth study of the level of food security in the Yangtze River Economic Belt: first, there is an overall upward trend in the level of food security, indicating that the Yangtze River Economic Belt has achieved remarkable progress in the area of food security. Second, there are significant differences in food security levels between regions, with relatively low food security indexes in the upstream regions and relatively high ones in the downstream regions. Third, the overall differences in food security levels in the Yangtze River Economic Belt are narrowing, and the differences within regions are also shrinking simultaneously; however, the problem of inter-regional imbalance persists. Fourth, the distribution dynamics of the food security level show an olive-shaped structure of “big in the middle and small at the ends”, with some polarization, but no stable multi-polarization has been formed yet. Fifth, there is a certain degree of convergence in the level of food security between regions, with lower initial-level regions experiencing a catch-up effect through the diffusion of technology and factor flows.

### 6.2 Policy recommendations

(1) Integrated policy responses to the multidimensional character of food security

In terms of quantitative security, we should continue to pay attention to core indicators such as food production and per capita production, adhere to the red line of arable land, resolutely curb the “de-farming” and “de-fooding” of arable land, and strengthen the construction of farmland water conservancy infrastructure to improve the ability of agriculture to withstand natural disasters. In terms of qualitative security, it is necessary to optimize the structure of agricultural production, and increase the supply of high-quality protein food based on guaranteeing food production; in terms of ecological security, it is necessary to actively promote ecologically friendly planting patterns, and pay attention to ecological environmental protection in the production process, to realize the coordinated development of food security and ecological security; in terms of capacity security, it is possible to increase the investment in rural infrastructure to improve the conditions of production and living in rural areas, and to improve the efficiency of agricultural production. In terms of capacity security, investment in rural infrastructure construction can be increased to enhance rural production and living conditions, as well as improve the efficiency of agricultural production.

(2) Promoting regional coordinated development

Strengthening industrial cooperation and synergistic development between the upstream, midstream and downstream regions of the Yangtze River Economic Belt, the downstream regions can utilize their technological, financial and market advantages to carry out cooperation with the midstream and upstream regions in the fields of agricultural product processing, circulation, etc., and to extend the grain industry chain to increase the added value of the grain industry to promote inter-regional industrial complementarities and resource-sharing.

(3) Regional policies for locally adapted development

The upstream areas can rationally develop and utilize mountainous agricultural resources under the premise of strengthening ecological protection, develop special food industries, and realize the benign interaction between food security and ecological protection; the midstream areas should give full play to the advantages of commodity grain bases, increase support for grain production, stabilize the grain planting area and improve the production capacity of grains; and the downstream areas should focus on the transformation and upgrading of agriculture and the construction of food security guarantee capacity. Downstream areas should focus on agricultural transformation and upgrading, as well as building food security capacity. This includes promoting food science and technology innovation, developing deep processing of food, and enhancing the competitiveness and added value of the food industry.
